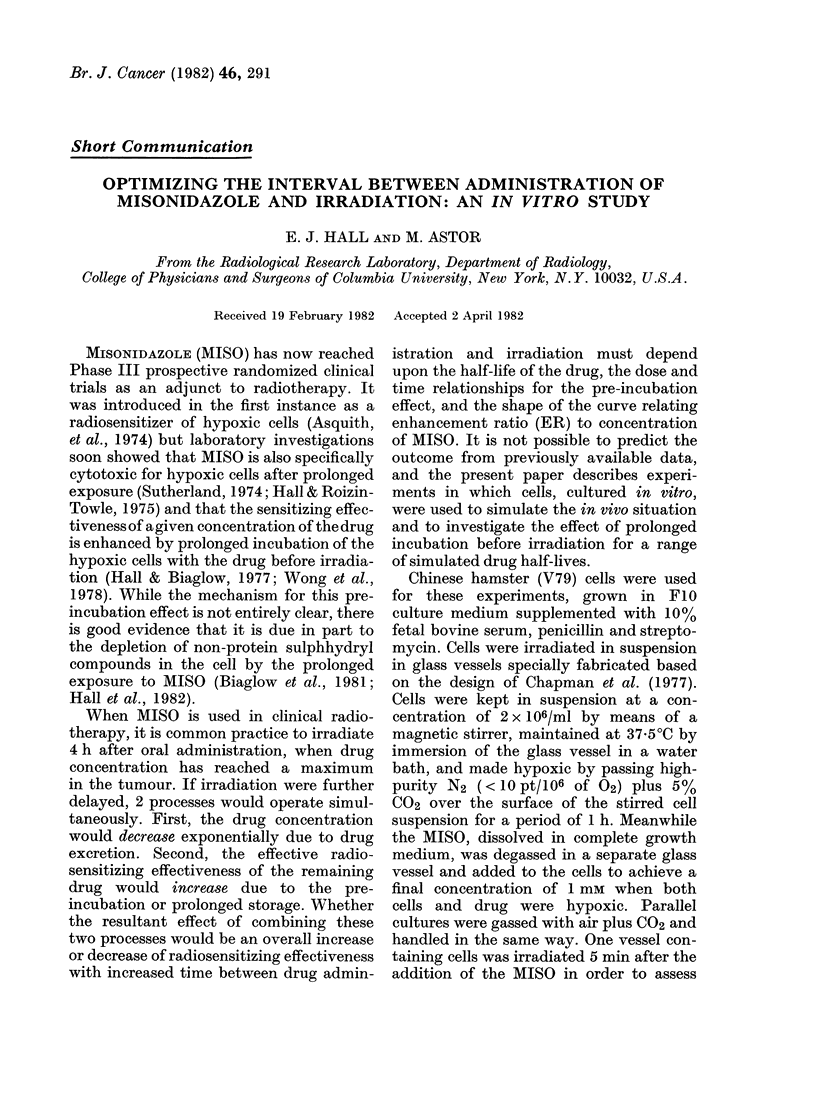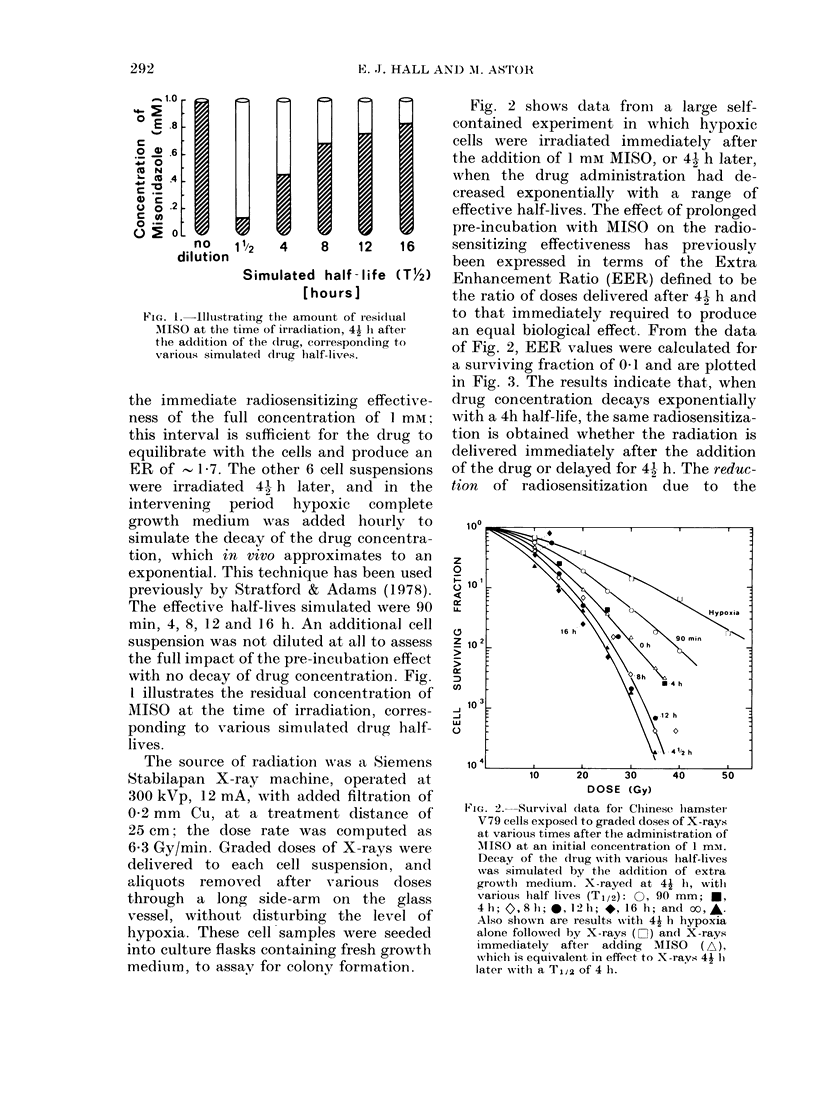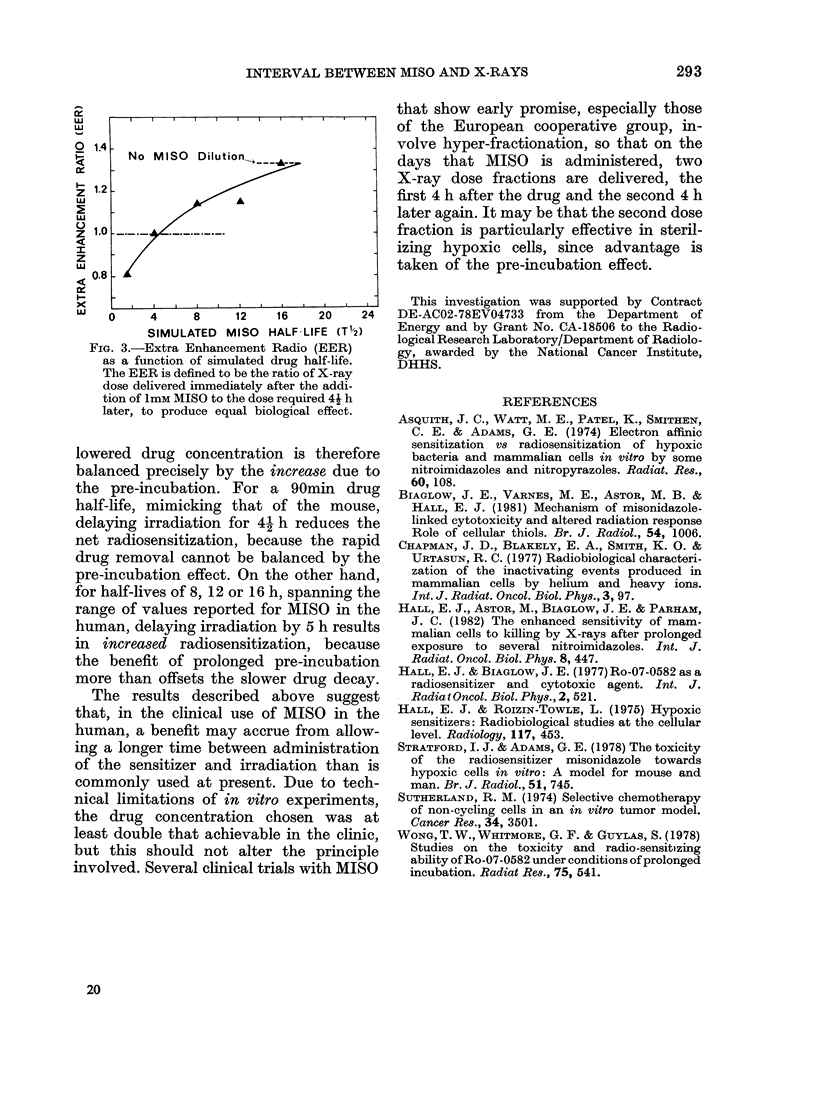# Optimizing the interval between administration of misonidazole and irradiation: an in vitro study.

**DOI:** 10.1038/bjc.1982.196

**Published:** 1982-08

**Authors:** E. J. Hall, M. Astor


*Br. J. Cancer* (1982) **46**, 291

***Short Communication***

### OPTIMIZING THE INTERVAL BETWEEN ADMINISTRATION OF MISONIDAZOLE AND IRRADIATION: AN *IN VITRO* STUDY

E. J. HALL AND M. ASTOR

*From the Radiological Research Laboratory, Department of Radiology, College of Physicians and Surgeons of Columbia University, New York, N.Y.* 10032, *U.S.A.*

Received 19 February 1982 Accepted 2 April 1982

MISONIDAZOLE (MISO) has now reached Phase III prospective randomized clinical trials as an adjunct to radiotherapy. It was introduced in the first instance as a radiosensitizer of hypoxic cells (Asquith, *et al.*, 1974) but laboratory investigations soon showed that MISO is also specifically cytotoxic for hypoxic cells after prolonged exposure (Sutherland, 1974; Hall & Roizin-Towle, 1975) and that the sensitizing effectiveness of a given concentration of the drug is enhanced by prolonged incubation of the hypoxic cells with the drug before irradiation (Hall & Biaglow, 1977; Wong *et al.*, 1978). While the mechanism for this pre-incubation effect is not entirely clear, there is good evidence that it is due in part to the depletion of non-protein sulphhydryl compounds in the cell by the prolonged exposure to MISO (Biaglow *et al.*, 1981; Hall *et al.*, 1982).

When MISO is used in clinical radiotherapy, it is common practice to irradiate 4 h after oral administration, when drug concentration has reached a maximum in the tumour. If irradiation were further delayed, 2 processes would operate simultaneously. First, the drug concentration would *decrease* exponentially due to drug excretion. Second, the effective radiosensitizing effectiveness of the remaining drug would *increase* due to the pre-incubation or prolonged storage. Whether the resultant effect of combining these two processes would be an overall increase or decrease of radiosensitizing effectiveness with increased time between drug administration and irradiation must depend upon the half-life of the drug, the dose and time relationships for the pre-incubation effect, and the shape of the curve relating enhancement ratio (ER) to concentration of MISO. It is not possible to predict the outcome from previously available data, and the present paper describes experiments in which cells, cultured *in vitro*, were used to simulate the *in vivo* situation and to investigate the effect of prolonged incubation before irradiation for a range of simulated drug half-lives.

Chinese hamster (V79) cells were used for these experiments, grown in F10 culture medium supplemented with 10% fetal bovine serum, penicillin and streptomycin. Cells were irradiated in suspension in glass vessels specially fabricated based on the design of Chapman *et al.* (1977). Cells were kept in suspension at a concentration of $2 \times 10^6$/ml by means of a magnetic stirrer, maintained at 37·5°C by immersion of the glass vessel in a water bath, and made hypoxic by passing high-purity $N_2$ (< 10 pt/$10^6$ of $O_2$) plus 5% $CO_2$ over the surface of the stirred cell suspension for a period of 1 h. Meanwhile the MISO, dissolved in complete growth medium, was degassed in a separate glass vessel and added to the cells to achieve a final concentration of 1 mM when both cells and drug were hypoxic. Parallel cultures were gassed with air plus $CO_2$ and handled in the same way. One vessel containing cells was irradiated 5 min after the addition of the MISO in order to assess

FIG. 1.—Illustrating the amount of residual MISO at the time of irradiation, $4\frac{1}{2}$ h after the addition of the drug, corresponding to various simulated drug half-lives.

the immediate radiosensitizing effectiveness of the full concentration of 1 mM; this interval is sufficient for the drug to equilibrate with the cells and produce an ER of $\sim 1{\cdot}7$. The other 6 cell suspensions were irradiated $4\frac{1}{2}$ h later, and in the intervening period hypoxic complete growth medium was added hourly to simulate the decay of the drug concentration, which *in vivo* approximates to an exponential. This technique has been used previously by Stratford & Adams (1978). The effective half-lives simulated were 90 min, 4, 8, 12 and 16 h. An additional cell suspension was not diluted at all to assess the full impact of the pre-incubation effect with no decay of drug concentration. Fig. 1 illustrates the residual concentration of MISO at the time of irradiation, corresponding to various simulated drug half-lives.

The source of radiation was a Siemens Stabilapan X-ray machine, operated at 300 kVp, 12 mA, with added filtration of 0·2 mm Cu, at a treatment distance of 25 cm; the dose rate was computed as 6·3 Gy/min. Graded doses of X-rays were delivered to each cell suspension, and aliquots removed after various doses through a long side-arm on the glass vessel, without disturbing the level of hypoxia. These cell samples were seeded into culture flasks containing fresh growth medium, to assay for colony formation.

Fig. 2 shows data from a large self-contained experiment in which hypoxic cells were irradiated immediately after the addition of 1 mM MISO, or $4\frac{1}{2}$ h later, when the drug administration had decreased exponentially with a range of effective half-lives. The effect of prolonged pre-incubation with MISO on the radiosensitizing effectiveness has previously been expressed in terms of the Extra Enhancement Ratio (EER) defined to be the ratio of doses delivered after $4\frac{1}{2}$ h and to that immediately required to produce an equal biological effect. From the data of Fig. 2, EER values were calculated for a surviving fraction of 0·1 and are plotted in Fig. 3. The results indicate that, when drug concentration decays exponentially with a 4h half-life, the same radiosensitization is obtained whether the radiation is delivered immediately after the addition of the drug or delayed for $4\frac{1}{2}$ h. The *reduction* of radiosensitization due to the

FIG. 2.—Survival data for Chinese hamster V79 cells exposed to graded doses of X-rays at various times after the administration of MISO at an initial concentration of 1 mM. Decay of the drug with various half-lives was simulated by the addition of extra growth medium. X-rayed at $4\frac{1}{2}$ h, with various half lives ($T_{1/2}$): ○, 90 mm; ■, 4 h; ◇, 8 h; ●, 12 h; ◆, 16 h; and ∞, ▲. Also shown are results with $4\frac{1}{2}$ h hypoxia alone followed by X-rays (□) and X-rays immediately after adding MISO (△), which is equivalent in effect to X-rays $4\frac{1}{2}$ h later with a $T_{1/2}$ of 4 h.

FIG. 3.—Extra Enhancement Radio (EER) as a function of simulated drug half-life. The EER is defined to be the ratio of X-ray dose delivered immediately after the addition of 1mM MISO to the dose required 4½ h later, to produce equal biological effect.

lowered drug concentration is therefore balanced precisely by the *increase* due to the pre-incubation. For a 90min drug half-life, mimicking that of the mouse, delaying irradiation for 4½ h reduces the net radiosensitization, because the rapid drug removal cannot be balanced by the pre-incubation effect. On the other hand, for half-lives of 8, 12 or 16 h, spanning the range of values reported for MISO in the human, delaying irradiation by 5 h results in *increased* radiosensitization, because the benefit of prolonged pre-incubation more than offsets the slower drug decay.

The results described above suggest that, in the clinical use of MISO in the human, a benefit may accrue from allowing a longer time between administration of the sensitizer and irradiation than is commonly used at present. Due to technical limitations of *in vitro* experiments, the drug concentration chosen was at least double that achievable in the clinic, but this should not alter the principle involved. Several clinical trials with MISO that show early promise, especially those of the European cooperative group, involve hyper-fractionation, so that on the days that MISO is administered, two X-ray dose fractions are delivered, the first 4 h after the drug and the second 4 h later again. It may be that the second dose fraction is particularly effective in sterilizing hypoxic cells, since advantage is taken of the pre-incubation effect.

This investigation was supported by Contract DE-AC02-78EV04733 from the Department of Energy and by Grant No. CA-18506 to the Radiological Research Laboratory/Department of Radiology, awarded by the National Cancer Institute, DHHS.

### REFERENCES

ASQUITH, J. C., WATT, M. E., PATEL, K., SMITHEN, C. E. & ADAMS, G. E. (1974) Electron affinic sensitization *vs* radiosensitization of hypoxic bacteria and mammalian cells *in vitro* by some nitroimidazoles and nitropyrazoles. *Radiat. Res.*, **60**, 108.

BIAGLOW, J. E., VARNES, M. E., ASTOR, M. B. & HALL, E. J. (1981) Mechanism of misonidazole-linked cytotoxicity and altered radiation response Role of cellular thiols. *Br. J. Radiol.*, **54**, 1006.

CHAPMAN, J. D., BLAKELY, E. A., SMITH, K. O. & URTASUN, R. C. (1977) Radiobiological characterization of the inactivating events produced in mammalian cells by helium and heavy ions. *Int. J. Radiat. Oncol. Biol. Phys.*, **3**, 97.

HALL, E. J., ASTOR, M., BIAGLOW, J. E. & PARHAM, J. C. (1982) The enhanced sensitivity of mammalian cells to killing by X-rays after prolonged exposure to several nitroimidazoles. *Int. J. Radiat. Oncol. Biol. Phys.* **8**, 447.

HALL, E. J. & BIAGLOW, J. E. (1977) Ro-07-0582 as a radiosensitizer and cytotoxic agent. *Int. J. Radiat Oncol. Biol. Phys.*, **2**, 521.

HALL, E. J. & ROIZIN-TOWLE, L. (1975) Hypoxic sensitizers: Radiobiological studies at the cellular level. *Radiology*, **117**, 453.

STRATFORD, I. J. & ADAMS, G. E. (1978) The toxicity of the radiosensitizer misonidazole towards hypoxic cells *in vitro*: A model for mouse and man. *Br. J. Radiol.*, **51**, 745.

SUTHERLAND, R. M. (1974) Selective chemotherapy of non-cycling cells in an *in vitro* tumor model. *Cancer Res.*, **34**, 3501.

WONG, T. W., WHITMORE, G. F. & GUYLAS, S. (1978) Studies on the toxicity and radio-sensitizing ability of Ro-07-0582 under conditions of prolonged incubation. *Radiat Res.*, **75**, 541.